# RadarSSM: A Lightweight Spatiotemporal State Space Network for Efficient Radar-Based Human Activity Recognition

**DOI:** 10.3390/s26072259

**Published:** 2026-04-06

**Authors:** Rubin Zhao, Fucheng Miao, Yuanjian Liu

**Affiliations:** 1 College of Electronic and Optical Engineering & College of Flexible Electronics (Future Technology), Nanjing University of Posts and Telecommunications, Nanjing 210023, China; 2018020229@njupt.edu.cn; 2 Graduate School of Science and Technology, Shinshu University, Nagano 390-8621, Japan; 25w6089k@shinshu-u.ac.jp

**Keywords:** millimeter-wave radar, human activity recognition, lightweight network, spatiotemporal decoupling, state space model

## Abstract

Millimeter-wave radar has gradually gained popularity as a sensor mode for Human Activity Recognition (HAR) in recent years because it preserves the privacy of individuals and is resistant to environmental conditions. Nevertheless, the fast inference of high-dimensional and sparse 4D radar data is still difficult to perform on low-resource edge devices. Current models, including 3D Convolutional Neural Networks and Transformer-based models, are frequently plagued by extensive parameter overhead or quadratic computational complexity, which restricts their applicability to edge applications. The present paper attempts to resolve these issues by introducing RadarSSM as a lightweight spatiotemporal hybrid network in the context of radar-based HAR. The explicit separation of spatial feature extraction and temporal dependency modeling helps RadarSSM decrease the overall complexity of computation significantly. Specifically, a spatial encoder based on depthwise separable 3D convolutions is designed to efficiently capture fine-grained geometric and motion features from voxelized radar data. For temporal modeling, a bidirectional State Space Model is introduced to capture long-range temporal dependencies with linear time complexity O(T), thereby avoiding the quadratic cost associated with self-attention mechanisms. Extensive experiments conducted on public radar HAR datasets demonstrate that RadarSSM achieves accuracy competitive with state-of-the-art methods while substantially reducing parameter count and computational cost relative to representative convolutional baselines. These results validate the effectiveness of RadarSSM and highlight its suitability for efficient radar sensing on edge hardware.

## 1. Introduction

### 1.1. Background and Motivation

Human Activity Recognition (HAR) has become a fundamental enabling technology for a wide range of applications, including elderly health monitoring, smart home automation and ambient assisted living [1,2]. With the rapid aging of the population and growing public health concerns, there is an increasing demand for continuous, non-intrusive and privacy-preserving activity-monitoring solutions [3,4]. Millimeter-wave (mmWave) radar is a promising option compared to vision-based methods, which tend to be more privacy sensitive [5], and wearable sensor systems that depend on user compliance and long-term wearability [6]. Wi-Fi channel state information-based sensing has also emerged as a non-intrusive HAR modality operating on existing infrastructure [7]; however, it is susceptible to multipath interference and lacks the fine-grained spatial resolution needed to distinguish subtle motion differences [8]. Its device-free nature and resilience to adverse conditions such as lighting changes, smoke and occlusion make mmWave radar especially suitable for private and complex in-home environments [9,10].

The large bandwidth and high carrier frequency nature of the mmWave radar make it possible to measure range, velocity and angular information on very fine scales, which helps to capture even subtle human motion patterns [9]. Nevertheless, these advantages come at the cost of data complexity. The radar measurements are commonly modeled as high-dimensional and sparse spatiotemporal sequences, particularly when they are voxelized to 4D representations. Real-time processing of such data streams on resource-limited edge devices creates a major computational and memory bottleneck [11]. Consequently, an important research issue will be finding a well-balanced approach between spatiotemporal modeling capabilities and computational efficiency, which will ensure precise and detailed HAR at the network edge. More broadly, lightweight architecture design has also become an important direction in RF deep learning tasks beyond HAR, such as automatic modulation classification, which further underscores the practical value of efficiency-aware model design for wireless sensing and inference [12].

### 1.2. Existing Methods and Limitations

To understand the behavior of humans through intricate radar reflections, recent works can be categorized into two supplementary areas, which are choosing a method of representing radar data and coming up with a design of deep learning architectures. Although significant advances have been achieved, there is still a challenge of effectively modelling long radar sequences in the presence of tight edge constraints.

#### 1.2.1. Diversity and Trade-Offs in Radar Data Representations

Raw radar data are often converted into intermediate representations to enable learning. Every representation adds different trade-offs between information preservation and the cost of computation.

- Micro-Doppler Spectrograms: Early radar-based HAR approaches focused on time-frequency analysis to produce the micro-Doppler spectrograms that are then analyzed with the aid of 2D Convolutional Neural Networks (CNNs). Despite being computationally efficient, they reduce three-dimensional spatial information to two-dimensional projections with the inevitable loss of range and angular signals. Therefore, in cases of restricted changes in Doppler such as in stationary or shifting movements, the recognition performance tends to decline [10,13].
- Sparse Point Clouds: Detection algorithm representations of point clouds, including Constant False Alarm Rate (CFAR), have a smaller data volume and are inherently sparse. Nevertheless, radar point clouds are usually unorganized, noisy and vulnerable to multipath clutter [14]. Irregularity in their structure makes it difficult to retrieve coherent geometric properties over time and due to limitations in hardware, they often end up with extremely sparse point clouds, which do not capture minor motion features [4]. More clustering or resampling operations need to be implemented, adding more complexity to the system and the preprocessing delay [9].
- Voxelized Sequences: In order to mitigate against the disorderliness of point clouds, voxelization subdivides radar data into uniform 4D lattices that allow using dense convolutional operators to extract spatial features. Although this representation enhances hardware compatibility, a rapid increase in computational load and memory usage can also be observed due to the fact that the expense of voxel-based processing scales sharply with spatial resolution [10].

#### 1.2.2. Evolution and Bottlenecks of Mainstream Architectures

The abovementioned data representations are matched by several different deep learning models. However, there are still some structural constraints when it comes to efficiently modeling long spatiotemporal radar sequences [10].

- 3D Convolutional Neural Networks (3D-CNNs): For voxelized radar data, 3D-CNNs are popular because they can both learn and use the fact that spatial correlation exists [9,15]. But the computational cost of the typical 3D convolution rises cubically with kernel size *K* ($O(K^{3})$), so it has a lot of redundancy. Besides, short temporal receptive fields prevent the learning of long-range activity contexts, which are required to identify full motion cycles [4].
- Graph Neural Networks (GNNs): The GNN-based methods can be applied to unstructured point clouds; however, the dynamic graph generation and information exchange have significant computing costs. Also, non-regular memory access patterns usually lead to less efficient inference rates when implemented on embedded hardware than conventional CNNs [9,16].
- Recurrent Neural Networks (RNNs): To model the temporal development, RNNs can sometimes be utilized to work with sequential radar features [17]. But their sequential computation nature restricts the use of parallelism in training and inference. Moreover, even with such gating mechanisms, RNNs find it hard to preserve the overall contextual knowledge over long radar sequences [13].
- Transformer Architectures: Transformer models utilize self-attention to learn global time-based interdependencies and have demonstrated a high potential in radar-based HAR [4,18]. However, the time and space complexity of their computation grows quadratically with the sequence length *T* ($O(T^{2})$) [19], which makes it difficult to run them on resource-constrained edge devices despite their high modeling power [9,10].

On the whole, the current methods have issues on how to keep spatial details, model long-range temporal dependencies with linear complexity and be efficient in terms of edge devices. In order to minimize the repetition of regular 3D convolutions, Depthwise Separable Convolution (DS-Conv3D) [20,21] offers a promising light solution to radar data processing. Meanwhile, recent advances in State Space Models (SSMs) [22,23] provide new opportunities for long-sequence modeling with linear complexity through selective scanning mechanisms. These observations collectively motivate the proposal of RadarSSM.

### 1.3. Main Contributions

To address the aforementioned challenges, this paper proposes RadarSSM, a novel lightweight spatiotemporal architecture tailored for resource-constrained edge radar sensing. The main contribution lies in the customized integration of DS-Conv3D and SSMs to systematically tackle the inherent sparsity and high dimensionality of 4D radar voxel data. This combination is non-trivial in two respects specific to mmWave radar: (1) DS-Conv3D’s channel-spatial decoupling acts as an implicit sparsity-aware regularizer, since standard 3D convolutions impose dense parameter tensors that are ill-matched to the sparse occupancy patterns of radar voxels—as confirmed by the 6× Multiply-Accumulate operations (MACs) increase and simultaneous accuracy degradation observed when DS-Conv3D is replaced with standard convolutions (Section 4.4); (2) beyond the well-known $O(T^{2})$ vs. O(T) complexity gap, SSMs model the smooth continuous state evolution characteristic of human activity sequences—gradual limb trajectories that carry temporal continuity—whereas self-attention treats all pairwise interactions symmetrically and lacks an inherent sequential state update mechanism.

The main contributions are summarized as follows:

1. Efficient spatial encoding for radar voxels: We adopt depthwise separable 3D convolution as the core spatial feature extractor to mitigate the computational redundancy of standard 3D convolutions. By decoupling spatial and channel correlations, the proposed encoder aligns with the sparsity characteristics of radar data, preserving fine-grained geometric features while significantly reducing computational cost.
2. SSM-based temporal modeling for radar HAR: To overcome the quadratic complexity of Transformer-based temporal modeling, we incorporate a bidirectional State Space Model (Bi-SSM) as the temporal module. Leveraging the linear O(T) complexity of SSMs [24], the proposed design captures non-causal long-range temporal dependencies within activity sequences, providing an efficient alternative to self-attention mechanisms.
3. System-level optimization for edge deployment: Extensive experiments on public radar HAR datasets demonstrate that RadarSSM achieves accuracy comparable to state-of-the-art Transformer-based methods while substantially reducing parameter count and computational cost relative to representative convolutional baselines, validating its suitability for real-time deployment on edge hardware.

## 2. Problem Formulation

In this section, RadarSSM is formulated as a spatiotemporal sequence-modeling problem. This process is decomposed into two cascaded sub-processes: frame-level spatial feature abstraction and long-range temporal state inference.

### 2.1. Input and Action Space

Consider a dataset $D={\left\{\left(X^{\left(i\right)},y^{\left(i\right)}\right)\right\}}_{i=1}^{N}$ consisting of *N* samples. To address the disorder and sparsity inherent in raw radar point clouds [9], the point cloud sequences are converted into a regular voxel representation. Consequently, an input sample X is represented as a sequence of radar voxel frames of length *T*, denoted as $X=\{x_{1},x_{2},\ldots ,x_{T}\}$, where $x_{t}\in R^{D\times H\times W}$ represents the voxel tensor at time *t* (Depth × Height × Width). The target action label *y* belongs to a discrete space {1,…,K}, where *K* is the total number of action classes.

### 2.2. Spatiotemporal Decomposition

In order to work with high-dimensional radar data when the available computing resources are restricted, the prediction function $f_{\theta }:X\to Y$ is mathematically broken down into two stages, namely spatial feature abstraction and temporal state inference.

#### 2.2.1. Spatial Feature Abstraction

The spatial mapping function $F_{\mathrm{spatial}}\left(\cdot ;\theta_{s}\right)$ is defined as processing the input sequence, where $\theta_{s}$ represents the learnable parameters of the spatial encoder. This function maps the data of every frame $x_{t}$ in high-dimensional voxel space into a lower dimensional latent feature space:

(1) $$z_{t}=F_{\mathrm{spatial}}\left(x_{t};\theta_{s}\right)\in R^{d_{\mathrm{model}}},$$

where $d_{\mathrm{model}}$ indicates the dimensions of the channel of the latent feature vectors. The original volumetric sequence X now becomes a compact series of feature vectors $Z=\{z_{1},z_{2},\ldots ,z_{T}\}$. This process attempts to achieve the maximum reduction of space redundancy without losing significant geometric motion data.

#### 2.2.2. Temporal State Evolution

After obtaining the feature sequence, HAR is represented as a process that evolves over time. To incorporate the historical context, one introduces the hidden state $h_{t}\in R^{d_{\mathrm{model}}}$ and defines the temporal development via the state update function:

(2) $$h_{t}=F_{\mathrm{state}}\left(h_{t-1},z_{t};\theta_{t}\right),\;t=1,\ldots ,T,$$

where $\theta_{t}$ denotes the learnable parameters of the temporal evolution.

To leverage global temporal context for sequence-level classification, we adopt a bi-directional strategy and use the last-step states from both directions as the sequence representation. Let $s_{\mathrm{fwd}}$ denote the last-step hidden state obtained by processing *Z* in the forward order, and $s_{\mathrm{bwd}}$ denote the last-step hidden state obtained by processing the reversed sequence. The aggregated representation is constructed as:

(3) $$h_{\mathrm{final}}=\mathrm{Concat}\left(s_{\mathrm{fwd}},\;s_{\mathrm{bwd}}\right)\in R^{2d_{\mathrm{model}}}.$$

Finally, the action probability prediction is produced by the classification head $F_{\mathrm{head}}$ parameterized by $\theta_{\mathrm{head}}$:

(4) $$\hat{y}=\mathrm{Softmax}\left(F_{\mathrm{head}}\left(h_{\mathrm{final}};\theta_{\mathrm{head}}\right)\right).$$

### 2.3. Optimization Objective

Training seeks the optimal parameter set $\theta =\{\theta_{s},\theta_{t},\theta_{\mathrm{head}}\}$ that minimizes the divergence between the predicted distribution and the true distribution. The cross-entropy loss function with Label Smoothing Regularization [25] is employed as the objective function:

(5) $$L\left(\theta \right)=-\frac{1}{B}\sum_{i=1}^{B}\sum_{k=1}^{K}y_{i,k}^{\mathrm{LS}}\log\left({\hat{y}}_{i,k}\right),$$

where *B* is the batch size, *K* is the number of action classes, ${\hat{y}}_{i,k}$ is the predicted probability for class *k* (the *k*-th element of vector ${\hat{y}}_{i}$), and $y_{i,k}^{\mathrm{LS}}$ denotes the smoothed ground truth label. The smoothed ground truth distribution $y_{i,k}^{\mathrm{LS}}$ is formulated as:

(6) $$y_{i,k}^{\mathrm{LS}}=\left(1-\epsilon \right)y_{i,k}+\frac{\epsilon }{K},$$

where $y_{i,k}$ denotes the original one-hot label and ϵ is the smoothing factor.

## 3. Our Proposed Method

**Figure 1:**
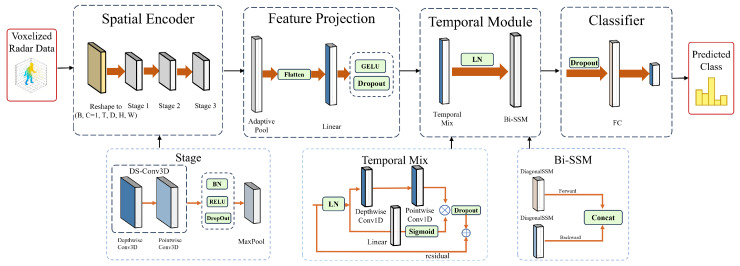
Overall architecture of the proposed RadarSSM.

This section details the proposed RadarSSM. Illustrated in Figure 1, the network is designed to achieve effective human action recognition from sparse, high-dimensional 4D radar point cloud voxel sequences within a limited computational budget. The detailed layer-wise network flow and the training procedure are presented in Table 1 and Algorithm 1, respectively.
**Algorithm 1:** The training procedure of the proposed RadarSSM.
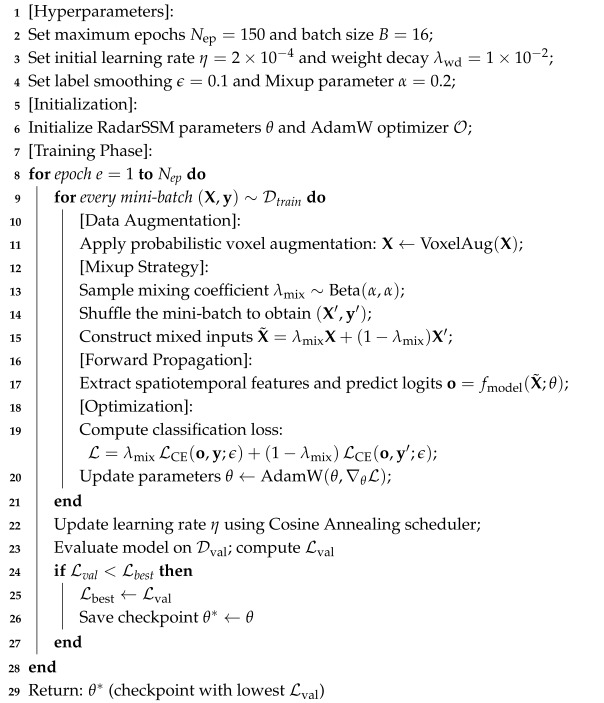


### 3.1. Overall Architecture

RadarSSM comprises three primary components designed to mitigate computational bottlenecks identified in existing literature [4]:

1. Efficient Spatial Encoder: Utilizes DS-Conv3D to extract local geometric and motion features from individual voxel frames.
2. Feature Projection: Reduces the dimensionality of high-dimensional spatial maps via adaptive pooling and projection.
3. Bi-Directional Hybrid Temporal Module: Merges a Local Temporal Mixer with a Bi-SSM. The mixer smooths local feature jitter, while the Bi-SSM captures long-range dependencies.

The choice to pair DS-Conv3D with a diagonal SSM rather than, for example, applying a Mamba backbone directly to flattened voxel sequences or augmenting DS-Conv3D with attention pooling is motivated by the geometric structure of radar data. A direct Mamba backbone would treat the spatial and temporal dimensions uniformly, discarding the separable range–azimuth–elevation geometry that DS-Conv3D exploits through its factored depthwise–pointwise decomposition. Conversely, pure DS-Conv3D with attention pooling would still incur $O(T^{2})$ cost for global temporal context and lacks the recurrent memory that smoothly accumulates activity state across a full 60-frame sequence.

### 3.2. Spatial Encoder

The voxel data of radar necessarily have a high dimensional 4D structure (T,D,H,W). Applying typical 3D convolutions directly leads to massive parameter redundancies and computational time. To overcome this, we build the spatial encoder as a stack of three cascaded blocks of DS-Conv3D, which are explicitly designed to decouple spatial and channel-wise feature extraction.

One singleton channel (C=1) is dynamically added to the beginning of the network. The resulting temporal dimension *T* is then merged with the batch dimension *B*, creating a tensor of size $X\in R^{(B\times T)\times 1\times D\times H\times W}$, and this enables the encoder to handle the voxel sequence as a sequence of frames. When the input tensor is $X_{\mathrm{in}}\in R^{(C_{\mathrm{in}}=1)\times D\times H\times W}$, the structure of DS-Conv3D is divided into two layers:

- Depthwise Conv3D: This applies spatial filtering on all the input channels independently with a K×K×K kernel to find the local geometric properties without altering the dimension of the channel.
- Pointwise Conv3D: This uses a 1×1×1 convolution to linearly combine spatially filtered outputs and fuses cross-channel information into new features.

Each DS-Conv3D stage is followed by Batch Normalization (BN), a ReLU activation and a MaxPool3D operation. Through this three-stage hierarchical extraction, the spatial dimensions are progressively reduced while the channel dimension is expanded. Finally, the processed features are reshaped back to the sequence format, yielding the output feature maps $F={\left\{F_{t}\right\}}_{t=1}^{T}$. Each frame $F_{t}\in R^{C^{\prime }\times D^{\prime }\times H^{\prime }\times W^{\prime }}$ denotes the compact spatial representation at time step *t*, where $C^{\prime }$ is the expanded channel dimension and $\left(D^{\prime },H^{\prime },W^{\prime }\right)$ are the downsampled spatial dimensions.

### 3.3. Feature Projection

The spatial encoder produces per-frame feature maps $F={\left\{F_{t}\right\}}_{t=1}^{T}$. To reduce the spatial redundancy and obtain compact tokens for temporal modeling, we apply a lightweight projection pipeline. Specifically, each $F_{t}$ is first downsampled by adaptive average pooling and then flattened to a $d_{m}$-dimensional vector:

(7) $${\tilde{f}}_{t}=\mathrm{Flatten}\left(\mathrm{AdaptiveAvgPool}3D(F_{t})\right)\in R^{d_{m}}.$$

Finally, a linear projection layer with GELU activation followed by dropout maps ${\tilde{f}}_{t}$ to the model dimension $d_{\mathrm{model}}$:

(8) $$z_{t}=\mathrm{Dropout}\left(\mathrm{GELU}\left(\mathrm{Linear}({\tilde{f}}_{t})\right)\right)\in R^{d_{\mathrm{model}}},$$

yielding the token sequence $Z={\left\{z_{t}\right\}}_{t=1}^{T}$ for the subsequent temporal module.

### 3.4. Temporal Module

Radar action sequences exhibit two distinct characteristics: short-term limb movements and long-term action evolution. A serial hybrid temporal module is designed to capture both simultaneously.

#### 3.4.1. Local Temporal Mixer

Prior to global modeling, a lightweight local mixing module is introduced to extract short-range features. To enhance feature selection, we adopt a gated convolutional design with a residual connection. The module consists of a Depthwise Separable 1D Convolution and a parallel gating branch. Given a feature sequence $Z\in R^{T\times d_{\mathrm{model}}}$, the mixer performs a sliding window operation to smooth feature jitter:

(9) $$Z_{\mathrm{local}}=Z+\mathrm{Dropout}\left(\text{DS-Conv1D}(\tilde{Z})⊙\sigma \left(\mathrm{Linear}\left(\tilde{Z}\right)\right)\right),$$

where $\tilde{Z}=\mathrm{LayerNorm}\left(Z\right)$, ⊙ denotes element-wise multiplication and σ is the Sigmoid function. A subsequent LayerNorm (LN) is applied before the SSM to stabilize the state transition:

(10) $$Z_{\mathrm{in}}=\mathrm{LayerNorm}\left(Z_{\mathrm{local}}\right).$$

#### 3.4.2. Bi-Directional SSM

Let $Z_{\mathrm{in}}=\left\{z_{1},z_{2},\ldots ,z_{T}\right\}$ denote the input sequence. Intuitively, the SSM operates as a learnable, input-dependent exponential moving average: at each step *t*, the decay gate $\alpha_{t}$ controls how much historical state is retained (values close to 1 imply long memory; close to 0 imply rapid forgetting), while the input gate $b_{t}⊙u_{t}$ injects new information weighted by the current token. The per-step step size $\Delta_{t}$ adapts this memory depth to the current input, enabling the model to selectively dwell on informative frames and skip over uninformative ones, while the recurrent formulation maintains a highly efficient O(T) linear complexity.

Given token $z_{t}\in R^{d_{\mathrm{model}}}$, we compute

(11) $$u_{t}=W_{u}z_{t},\;\Delta_{t}=\sigma \left(W_{\Delta }z_{t}\right),\;\left[b_{t},c_{t}\right]=W_{bc}z_{t},$$

where $W_{u},W_{\Delta },W_{bc}$ are learnable projection matrices. Specifically, $\Delta_{t}\in {\left(0,1\right)}^{d_{\mathrm{model}}}$ discretizes the continuous-time state space: a larger $\Delta_{t}$ corresponds to a longer effective time step, allowing selective emphasis on key temporal positions [23]. We subsequently apply gating $b_{t}\leftarrow \tanh\left(b_{t}\right)$ and $c_{t}\leftarrow \sigma \left(c_{t}\right)$.

The diagonal state transition rate a is derived from a learnable parameter $A_{\log}\in R^{d_{\mathrm{model}}}$ via $a=-\exp(A_{\log})$. This parameterization enforces the negativity of a, ensuring stable state decay. The time-varying decay gate is then computed as:

(12) $$\alpha_{t}=\exp\left(a⊙\Delta_{t}\right).$$

The −exp(·) parameterisation of a enforces $\alpha_{t}\in {\left(0,1\right)}^{d_{\mathrm{model}}}$, mathematically guaranteeing bounded state norms and numerical stability throughout the sequence.

The per-step state update and output are formulated as a gated state update:

(13) $$s_{t}=\alpha_{t}⊙s_{t-1}+\left(1-\alpha_{t}\right)⊙\left(b_{t}⊙u_{t}\right),\;y_{t}=c_{t}⊙s_{t}+D⊙u_{t},$$

where $u_{t}$ is the projected input, $y_{t}$ is the step output, and $D\in R^{d_{\mathrm{model}}}$ is a learnable skip vector. The skip connection via D provides a direct input path, preventing gradient vanishing for short-range temporal patterns.

As standard SSMs are causally strict, our strategy in the case of non-causal action recognition is to use a bi-directional system, inspired by Vision Mamba [24]. Importantly, the forward and backward branches maintain *independent* parameter sets $\left\{W_{u}^{\mathrm{fwd}},W_{\Delta }^{\mathrm{fwd}},W_{bc}^{\mathrm{fwd}},A_{\log}^{\mathrm{fwd}},D^{\mathrm{fwd}}\right\}$ and $\left\{W_{u}^{\mathrm{bwd}},\ldots ,D^{\mathrm{bwd}}\right\}$, respectively. This unshared design is critical, as it allows the model to learn asymmetric temporal patterns where the onset and conclusion phases of an activity carry distinct discriminative signatures. Precisely, we compute the SSM in the forward direction using $Z_{\mathrm{in}}$ in order to derive the last hidden state $s_{\mathrm{fwd}}$, and in the backward direction to derive the final backward state $s_{\mathrm{bwd}}$. In contrast to other ways that average all the outputs in a sequence, we use such final states explicitly as indicators of the overall context. The last temporal representation is formed by concatenation:

(14) $$h_{\mathrm{final}}=\mathrm{Concat}\left(s_{\mathrm{fwd}},s_{\mathrm{bwd}}\right)\in R^{2d_{\mathrm{model}}}.$$

This mechanism has a beneficial effect in compressing the entire activity history into a compact vector, which increases the robustness of the classification.

### 3.5. Classification Head

Classification is performed directly using the accumulated feature vector $h_{\mathrm{final}}$ from this Bi-SSM. To avoid overfitting, it is passed through a Dropout layer and transformed into the probability distributions of *K* action classes by a fully connected layer (FC). Such a structure prevents the possible information loss due to extra global average pooling layers.

## 4. Experimental Results and Analysis

### 4.1. Datasets

In order to assess the results of the application of RadarSSM, two publicly available mmWave radar point cloud datasets, namely, MMActivity and a part of MiliPoint were used to perform the experiments.

MMActivity [26]: These benchmark data were gathered through a TI IWR1443BOOST FMCW radar. There are five different exercises (Walking, Jumping, Jumping Jacks, Squats and Boxing) that were done by two subjects. There are 12,097 samples in the training set and 3538 samples in the test set. To build models, the training set was also divided into 9677 training examples and 2420 validation examples (8:2 proportion).

MiliPoint [27]: Using the MiliPoint dataset measured with a TI IWR1843 radar, it is possible to evaluate performance on more diverse conditions. In order not to have an inconsistent basis of assessment, a specific subset consisting of the first five unique activities—marching with bent arms (Marching), bent-arm chest expansion with side stepping (Chest Exp), straight-arm raises with side stepping (Arm Raise), bent-arm raises with left knee lift (L-Knee) and bent-arm raises with right knee lift (R-Knee)—was chosen to work with. This subset contains 2822 training examples, 314 validation examples and 406 test examples.

### 4.2. Implementation Details

Data Preprocessing: Voxelization converts the raw point cloud into a regular 4D grid with dimensions T×D×H×W=60×10×32×32, where *D*, *H* and *W* correspond to the depth (range), height (elevation) and width (azimuth) axes of the physical Region of Interest, respectively. Instead of a simple binary occupancy, the scalar value of each voxel directly encodes the spatial density of the radar reflections; specifically, it represents the absolute count of radar points falling within that 3D spatial boundary at time step *t*. Furthermore, to effectively mitigate the inherent sparsity of mmWave radar point clouds, points from two adjacent temporal frames are accumulated prior to voxelization. Consequently, empty voxels are assigned a value of 0, while active voxels contain positive integers reflecting the local density of human body reflections. This specific configuration was determined empirically to achieve a practical trade-off between recognition accuracy and computational overhead [26]. This is followed by adding a channel dimension (C=1), resulting in $X\in R^{B\times T\times 1\times D\times H\times W}$.

Training Configuration: Experiments were conducted on a workstation that has an Intel Core Ultra 9 CPU, 128GB of RAM and an NVIDIA GeForce RTX 5070 Ti. The models were written in PyTorch 2.8.0. The model checkpoint with the lowest validation loss across all 150 epochs was selected for final evaluation. No learning rate warm-up or early stopping was applied; training ran for the full 150 epochs in all experiments. Detailed hyperparameters are detailed in Table 2.

Regularization Strategy: In order to reduce the effect of overfitting with sparse radar observations, a composite regularization strategy was used. These are (1) Label Smoothing (ϵ=0.1); (2) a hierarchical Dropout scheme that is applied to the spatial encoder, temporal module and projection/classification heads (rates summarised in Table 2); and (3) augmentation at the level of data using Mixup (α=0.2) [28] and VoxelAug (Specifically, VoxelAug performs random circular spatial shifting to simulate subject displacement and temporal frame masking (random frame dropout) to improve robustness against intermittent sensor data loss [29].).

Reproducibility: The outcomes are expressed in terms of the average of 5 independent runs with varying random seeds. The training and validation splits had been randomized per seed, but the test set was held constant to make the comparisons fair. To additionally examine statistical robustness on the smaller MiliPoint dataset, training strategy variants are evaluated over N=10 independent runs in Section 4.4.

### 4.3. Comparative Analysis with State-of-the-Art Models

Table 3 presents a quantitative evaluation of the proposed RadarSSM against representative baseline models.

(1) Accuracy and Robustness: RadarSSM achieves 76.26 ± 2.59% on the MiliPoint dataset, substantially outperforming the second-best CNN-LSTM baseline (71.28 ± 5.46%), and 92.74 ± 0.90% on MMActivity, surpassing CNN-Transformer (91.27 ± 0.84%) and CNN-LSTM (91.03 ± 0.77%). It is essential to note that RadarSSM achieves these results at a substantially smaller parameter footprint than the baselines, which demonstrates a good trade-off between model complexity and performance. Moreover, the consistently low Expected Calibration Error (ECE) values (0.09 on MiliPoint and 0.07 on MMActivity) indicate reliable confidence calibration.

(2) Computational Efficiency: In terms of resource utilization, RadarSSM proves more efficient. As shown in Table 3, the model requires only 87.1 MMACs per inference. It is a significant decrease in the amount of computation required in comparison with other baseline models. Although the ultra-lightweight baselines like 1D-CNN (40.6 MMACs) and Bi-LSTM (43.4 MMACs) have smaller operation counts, they cannot compete on par with recognition accuracy. RadarSSM has effectively balanced this trade-off and can operate at high precision with a minimal parameter footprint of about 108.3 K.

(3) Statistical Significance: To rigorously assess the reliability of the performance margins, Table 4 reports per-seed accuracy, standard deviation and Welch’s *t*-test results for the top-performing baselines. On MiliPoint, RadarSSM achieves a higher mean accuracy than CNN-LSTM (76.26% vs. 71.28%) with a smaller standard deviation (2.59 vs. 5.46), although the corresponding *p*-value (0.115) indicates that this margin should be interpreted cautiously given the limited subset size. CNN-Transformer exhibits both a lower mean accuracy and larger variability. On MMActivity, RadarSSM maintains the highest mean accuracy, and its margins over CNN-LSTM (p=0.013) and CNN-Transformer (p=0.029) are statistically significant.

### 4.4. Ablation Study

An ablation study is performed to test the contribution of individual components, which is described in Table 5.

(1) Impact of Architecture Components: The deletion of the Bi-SSM module is associated with severe deterioration in performance, and accuracy decreases to 65.08% on MiliPoint and 91.07% on MMActivity. It confirms an essential contribution of SSM in the representation of long-range temporal correlations. In turn, elimination of the Temporal Mixer causes a decrease in accuracy, confirming the utility of Temporal Mixer in reducing the local feature jitter. It is worth noting that the use of standard 3D convolutions instead of the DS-Conv3D increases the computational cost by more than 5 times (87.1 MMACs as opposed to 536.3 MMACs). With this change goes an even sharper drop in accuracy (e.g., 89.26% on MMActivity and 70.49% on MiliPoint), indicating that DS-Conv3D is not merely efficient but rather also serves as a useful regularizer that avoids overfitting when using sparse voxel information.

To further validate the specific structural synergy, two alternative paradigms are evaluated. Replacing the SSM temporal module with attention-based global pooling (DS-Conv3D + AttnPooling) yields only 58.42±3.24% on MiliPoint and 90.61±1.22% on MMActivity (p<0.001 and p=0.016 respectively), confirming that $O(T^{2})$ attention pooling is both computationally inferior and less effective for capturing long-range activity dynamics. Applying a Direct Voxel-Mamba backbone to flattened per-frame features achieves 60.19±1.98% on MiliPoint and 87.66±0.43% on MMActivity (p<0.001 for both), despite its larger parameter count (1.08M). This validates the design rationale in Section 3.1: a monolithic sequence model cannot compensate for the loss of structured spatial encoding, and the two-stage decoupled design of RadarSSM is essential.

(2) Impact of Training Strategies: The integration of VoxelAug and Mixup further improves the proposed model on MMActivity. Starting from the vanilla baseline of 91.78%, applying VoxelAug or Mixup individually increases the accuracy to 92.27% and 92.32%, respectively, while their combination yields the best result of 92.74%.

On the smaller MiliPoint subset, the effect is more nuanced. The vanilla baseline achieves 74.68±4.40% accuracy, whereas applying VoxelAug or Mixup individually reduces the mean accuracy to 73.25±1.81% and 71.58±4.31%, respectively. We interpret this behavior as a small-data regularization-balance effect. Under this low-data regime, VoxelAug broadens the spatial–temporal support of sparse voxel patterns, while Mixup generates interpolated samples across class boundaries; when only 2822 training samples are available, either effect alone can become overly aggressive and degrade generalization. By contrast, combining the two strategies yields 76.26±2.59%, which is the best 5-run mean among the compared training strategies. In particular, the combined setting outperforms VoxelAug in 5/5 seeds and Mixup in 4/5 seeds, with mean gains of approximately +3.0 and +4.7 percentage points, respectively. This suggests that VoxelAug increases within-class diversity, whereas Mixup smooths the decision boundary through vicinal interpolation, and their combination acts more complementarily than either strategy alone.

To assess whether this trend is statistically robust, Table 6 further reports an extended evaluation over N=10 independent runs on MiliPoint. The 10-run results confirm that RadarSSM remains stronger in mean accuracy than the single-augmentation settings (74.66% vs. 72.51% for VoxelAug only and 72.02% for Mixup only), while also showing lower variability than the vanilla and Mixup-only settings. At the same time, the 10-run analysis does not support a statistically significant superiority over the vanilla baseline under Welch’s *t*-test. Therefore, the combined strategy is better interpreted as providing a more balanced and reliable trend than either single augmentation, rather than as a universally superior replacement for the vanilla setting on this sparse dataset.

### 4.5. Visualization Analysis

**Figure 2:**
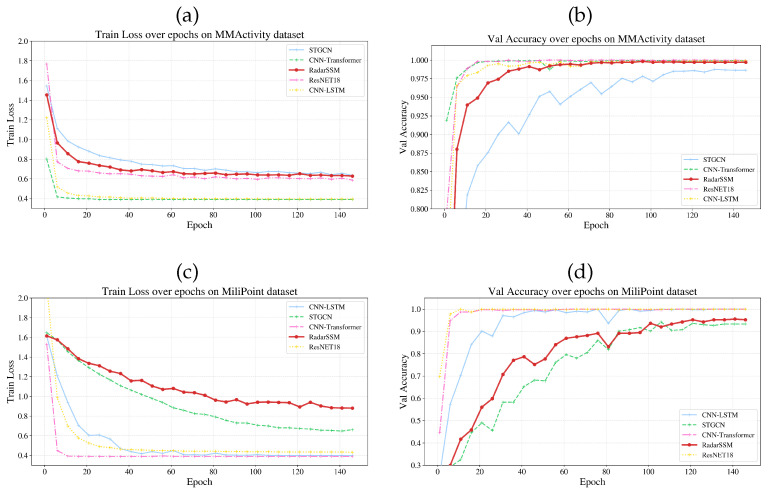
Training loss and validation accuracy comparisons of different models on the MiliPoint and MMActivity datasets. (**a**) Top-left: MMActivity Train Loss. (**b**) Top-right: MMActivity Val Acc. (**c**) Bottom-left: MiliPoint Train Loss. (**d**) Bottom-right: MiliPoint Val Acc.

**Figure 3:**
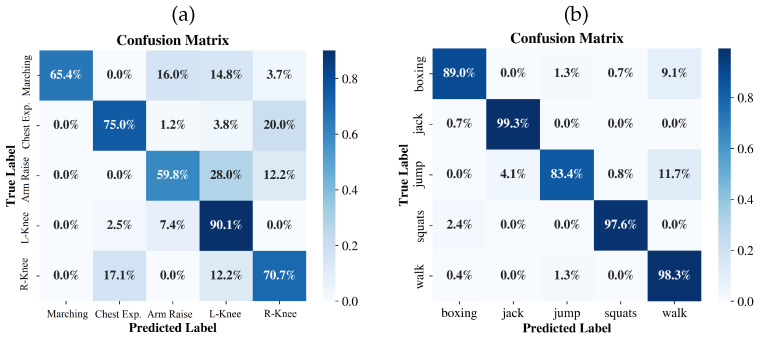
Confusion matrices of RadarSSM on the test sets. (**a**) Left: MiliPoint dataset. (**b**) Right: MMActivity dataset.

Figure 2 illustrates the training loss and validation accuracy curves. Unlike CNN-based baselines, which also have a sharp saturation trend that peaks within the first ten epochs, RadarSSM has a gradual and steady convergence pattern. The model has reached a higher validation accuracy (>90%) in less than 20 epochs on the MMActivity dataset, as well as a relatively constant optimization curve with slight fluctuations. The training loss in training RadarSSM decreases steadily and smoothly over longer periods on the more sophisticated MiliPoint dataset, which is an opposite behavior in comparison to the earlier stalling of the baseline methods. Figure 3 shows the confusion matrices to add more information on the results of recognizing the classes. The model has excellent precision on the MMActivity dataset (Figure 3b) and it can be noted that the three activities, i.e., Jumping Jacks, Squats and Walking, have achieved above 97% accuracy. There is a slight misclassification of “Jumping” (83.4%), which is mainly confused with “Walking” (11.7%).

On the more challenging MiliPoint dataset (Figure 3a), bent-arm raises with left knee lift achieves the highest recognition rate (90.1%). However, other categories exhibit distinct confusion patterns. Straight-arm raises with side stepping prove the most difficult to classify (59.8%) and is heavily misclassified as bent-arm raises with left knee lift (28.0%). Marching with bent arms (65.4%) also shows ambiguity, being confused with straight-arm raises (16.0%) and left knee lifts (14.8%). Furthermore, a significant mutual confusion is observed between bent-arm chest expansion (75.0%) and bent-arm raises with right knee lift (70.7%), where 20.0% of chest expansion samples are mislabeled as right knee lifts, and 17.1% of right knee lift samples are mislabeled as chest expansion. This suggests these pairs share highly similar spatiotemporal signatures in the sparse radar domain, particularly regarding the bent-arm dynamics.

## 5. Conclusions

This paper proposes RadarSSM, a lightweight spatiotemporal hybrid network for radar-based HAR on resource-constrained edge devices. By combining a DS-Conv3D spatial encoder with a bi-directional state-space temporal module, RadarSSM reduces the computational burden of voxel-sequence modeling while maintaining competitive recognition accuracy. Experimental results on MMActivity and MiliPoint show that RadarSSM achieves a favorable accuracy–efficiency trade-off with a compact footprint (108.3 K parameters and 87.1 MMACs per inference), reaching 92.74 ± 0.90% accuracy on MMActivity and 76.26 ± 2.59% on MiliPoint. Ablation results further indicate that removing the Bi-SSM leads to the largest accuracy drop, while replacing DS-Conv3D with standard 3D convolution substantially increases computation and degrades accuracy, confirming that depthwise separable spatial encoding is key for efficient deployment. RadarSSM adopts data augmentation and regularization (Mixup and VoxelAug) without channel-attention modules, yielding a stable accuracy-efficiency balance suitable for real-time deployment. Future work will focus on (1) extending the framework to multi-person and occlusion-heavy scenarios; (2) improving cross-domain robustness under environmental shifts via domain adaptation; (3) accelerating on-device inference through hardware-aware optimization such as quantization and operator-level; and (4) evaluating on larger-scale and more diverse radar HAR benchmarks, including multi-subject, multi-environment datasets, to further assess cross-domain generalization.

## Figures and Tables

**Table 1:** Network flow of the proposed RadarSSM.

		Output Tensor Shape
Stage	Operation Flow (Layer Configuration)	(*T* × *C* × *D* × *H* × *W*)
Input	Raw Voxel Sequence	60×1×10×32×32
Spatial Encoder	Processing per frame via DS-Conv3D	
Stage 1	DS-Conv3D (3×3×3) → BN → ReLU → MaxPool (2×2×2)	60×8×5×16×16
Stage 2	DS-Conv3D (3×3×3) → BN → ReLU → MaxPool (2×2×2)	60×16×2×8×8
Stage 3	DS-Conv3D (3×3×3) → BN → ReLU → MaxPool (1×2×2)	60×32×2×4×4
Feature Projection	Spatial-to-Feature Flattening	(Time × Features)
Projection	AdaptivePool (1,2,2)→ Flatten → Linear → GELU → Dropout	60×96
Temporal Module	Sequence Modeling	
Local Mixer	LayerNorm → Gated DS-Conv1D → Residual → LayerNorm	60×96
Bi-SSM	Bi-SSM → Concat	192 (Aggregated State)
Classifier	Action Prediction	
Head	Dropout (p=0.3) → Linear (Fully Connected)	*K* Classes (5)

**Table 2:** Experimental parameter settings.

Parameter	Value
Scheduler	CosineAnnealingLR
Loss function	CrossEntropyLoss
Label smoothing	0.1
Mixup parameter (α)	0.2
Learning rate	$2\times 10^{-4}$
Weight decay	0.01
Batch size	16
Epochs	150
Dropout (Spatial Encoder)	0.05
Dropout (Temporal Module)	0.10
Dropout (Projection & Head)	0.30

**Table 3:** Quantitative results of representative baseline models on both datasets.

Model	#Params	Acc (%)	Prec (%)	Rec (%)	F1-w (%)	F1-m (%)	MMACs	ECE
Panel A: Results on the MiliPoint Dataset								
ST-GCN [30]	170.0 K	38.03	41.26	38.03	35.09	35.13	1422.8	0.32
ResNet-18	295.1 K	45.66	48.69	45.66	44.72	44.74	889.7	0.10
Bi-LSTM	722.8 K	54.33	55.82	54.33	53.98	54.06	43.4	0.28
MLP	747.7 K	55.27	56.48	55.27	52.67	52.78	44.3	0.10
1D-CNN	697.8 K	56.21	58.23	56.21	53.65	53.77	40.6	0.14
CNN-Transformer	365.5 K	57.78	60.56	57.78	57.73	57.73	564.7	0.24
CNN-LSTM	216.9 K	71.28	75.17	71.28	70.72	70.75	561.1	0.12
**RadarSSM**	**108.3 K**	**76.26**	**78.12**	**76.26**	**75.90**	**75.94**	**87.1**	**0.09**
Panel B: Results on the MMActivity Dataset								
ST-GCN [30]	170.0 K	82.05	83.16	82.05	81.57	81.89	1422.8	0.04
1D-CNN	697.8 K	86.63	87.56	86.63	86.39	86.67	40.6	0.09
ResNet-18	295.1 K	87.08	87.97	87.08	86.74	86.97	889.7	0.14
MLP	747.7 K	88.52	89.43	88.52	88.28	88.53	44.3	0.12
Bi-LSTM	722.8 K	89.23	90.30	89.23	89.10	89.37	43.4	0.03
CNN-LSTM	216.9 K	91.03	92.39	91.03	90.98	91.26	561.1	0.02
CNN-Transformer	365.5 K	91.27	91.81	91.27	91.20	91.41	564.7	0.03
**RadarSSM**	**108.3 K**	**92.74**	**93.35**	**92.74**	**92.70**	**92.88**	**87.1**	**0.07**

Note: All reported metrics are averaged over 5 runs. #Params: Number of parameters; Acc: Accuracy; Prec: Precision; Rec: Recall; F1-m: F1-macro; ECE: Expected Calibration Error. MMACs: Million Multiply-Accumulate operations per inference. Best results in accuracy metrics are bolded. All baseline architectures are adapted lightweight versions tailored to ensure a fair comparison.

**Table 4:** Statistical significance analysis of accuracy on both datasets.

Model	Accuracy on Individual Seeds (%)					Avg. Acc.	Std. Dev.	95% CI ^‡^	*p*-Value ^†^
	R1	R2	R3	R4	R5				
Panel A: MiliPoint Dataset									
CNN-Transformer	60.34	61.08	51.23	49.26	67.00	57.78	7.38	[48.62,66.94]	0.003
CNN-LSTM	73.89	61.58	72.91	74.63	73.40	71.28	5.46	[64.50,78.06]	0.115
**RadarSSM**	75.12	79.31	78.08	72.66	76.11	**76.26**	**2.59**	[73.04,79.48]	–
Panel B: MMActivity Dataset									
CNN-Transformer	90.31	91.35	92.48	90.67	91.55	91.27	0.84	[90.23,92.31]	0.029
CNN-LSTM	90.70	92.11	90.05	91.41	90.90	91.03	0.77	[90.07,91.99]	0.013
**RadarSSM**	93.41	92.88	93.33	92.88	91.18	**92.74**	**0.90**	[91.62,93.86]	–

Note: ^‡^ The 95% Confidence Intervals (CI) are estimated based on the *t*-distribution with 4 degrees of freedom. ^†^ The *p*-values are calculated using two-tailed Welch’s *t*-test comparing each baseline model against our proposed RadarSSM, based on 5 independent runs with varying random seeds.

**Table 5:** Ablation study of key components, strategies and alternative paradigms.

Model Variant	#Params	Acc (%)	Std. Dev.	95% CI ^‡^	*p*-Value ^†^	MMACs
Panel A: MiliPoint Dataset						
Impact of Architecture Components						
w/o Temporal Mixer	89.1 K	69.71	2.29	[66.87,72.55]	0.003	86.0
w/o SSM	33.7 K	65.08	8.00	[55.15,75.01]	0.041	82.7
w/o Depthwise Conv	124.5 K	70.49	4.74	[64.60,76.38]	0.054	536.3
Alternative Architectural Designs						
DS-Conv3D + AttnPooling	19.4 K	58.42	3.24	[54.40,62.44]	<0.001	81.9
Direct Voxel-Mamba	1.08M	60.19	1.98	[57.73,62.65]	<0.001	65.9
Impact of Training Strategy						
Baseline (Vanilla)	108.3 K	74.68	4.40	[69.22,80.14]	0.513	87.1
w/Voxel Augmentation	108.3 K	73.25	1.81	[71.00,75.50]	0.076	87.1
w/Mixup	108.3 K	71.58	4.31	[66.23,76.93]	0.083	87.1
**RadarSSM (w/Mixup and VoxelAug)**	**108.3 K**	**76.26**	**2.59**	[73.04,79.48]	–	**87.1**
Panel B: MMActivity Dataset						
Impact of Architecture Components						
w/o Temporal Mixer	89.1 K	91.76	1.08	[90.42,93.10]	0.156	86.0
w/o SSM	33.7 K	91.07	1.07	[89.74,92.40]	0.033	82.7
w/o Depthwise Conv	124.5 K	89.26	0.43	[88.73,89.79]	<0.001	536.3
Alternative Architectural Designs						
DS-Conv3D + AttnPooling	38.5 K	90.61	1.22	[89.10,92.12]	0.016	81.9
Direct Voxel-Mamba	1.08M	87.66	0.43	[87.13,88.19]	<0.001	65.9
Impact of Training Strategy						
Baseline (Vanilla)	108.3 K	91.78	0.69	[90.92,92.64]	0.093	87.1
w/Voxel Augmentation	108.3 K	92.27	0.84	[91.23,93.31]	0.419	87.1
w/Mixup	108.3 K	92.32	1.05	[91.02,93.62]	0.518	87.1
**RadarSSM (w/Mixup and VoxelAug)**	**108.3 K**	**92.74**	**0.90**	** [91.62,93.86] **	–	**87.1**

Note: #Params: Number of parameters; Acc: Accuracy (mean, *N* = 5). MMACs: Million Multiply-Accumulate operations per inference. w/o: without specific module. ^‡^ The 95% Confidence Intervals (CI) are estimated based on the *t*-distribution with 4 degrees of freedom. ^†^ The *p*-values are computed using the two-tailed Welch’s *t*-test comparing each ablation variant against the final RadarSSM. Direct Voxel-Mamba and DS-Conv3D + AttnPooling represent the alternative paradigms evaluated to support the proposed architectural rationale.

**Table 6:** Statistical Significance analysis of training strategies on MiliPoint dataset.

Training Strategy	Accuracy on Individual Seeds (%)										Avg. Acc.	Std. Dev.	95% CI ^‡^	*p*-Value ^†^
	R1	R2	R3	R4	R5	R6	R7	R8	R9	R10				
Baseline (Vanilla)	73.65	79.06	78.57	68.23	73.89	67.49	74.88	79.56	77.83	79.80	75.30	4.54	[72.05,78.55]	0.717
w/VoxelAug	74.14	73.40	72.17	70.94	75.62	68.72	69.95	68.72	73.15	78.33	72.51	3.08	[70.31,74.71]	0.136
w/Mixup	69.46	72.41	78.57	70.20	67.24	67.98	67.73	82.02	73.89	70.69	72.02	4.90	[68.51,75.53]	0.170
RadarSSM ^*^	75.12	79.31	78.08	72.66	76.11	73.15	70.20	70.69	73.89	77.34	**74.66**	**3.08**	[72.46,76.86]	–

Note: ^*^ RadarSSM denotes the complete model trained with the combination of both VoxelAug and Mixup. ^‡^ The 95% Confidence Intervals (CI) are estimated based on the *t*-distribution with 9 degrees of freedom. ^†^ The *p*-values are calculated using the two-tailed Welch’s *t*-test comparing each ablation variant against our combined model based on 10 independent runs. Note that the vanilla baseline occasionally achieves high peak accuracy but also exhibits larger variability (±4.54). RadarSSM shows a lower standard deviation than the vanilla and Mixup-only settings and a higher mean accuracy than both single-augmentation variants, suggesting a more balanced trend on this sparse dataset. However, the current 10-run Welch’s *t*-test does not establish statistically significant superiority in mean accuracy over the vanilla baseline.

## Data Availability

The datasets generated and/or analyzed during the current study are available in the public repositories referenced in this paper (MMActivity [26] and MiliPoint [27]).
